# Hepatic outcomes among adults taking duloxetine: a retrospective cohort study in a US health care claims database

**DOI:** 10.1186/s12876-015-0373-4

**Published:** 2015-10-14

**Authors:** Nancy D. Lin, Heather Norman, Arie Regev, David G. Perahia, Hu Li, Curtis Liming Chang, David D. Dore

**Affiliations:** 1 Optum Epidemiology, Waltham, MA USA; 2 Eli Lilly and Company, Indianapolis, IN USA; 3 Eli Lilly and Company, Windlesham, Surrey UK; 4 Eli Lilly and Company, and Ditmanson Medical Foundation Chiayi Christian Hospital, Chiayi City, Taiwan; 5 Departments of Health Services, Policy, & Practice, and Epidemiology, Brown University School of Public Health, Providence, RI USA

**Keywords:** Duloxetine, Hepatic injury, Safety, Pharmacoepidemiology

## Abstract

**Background:**

Hepatic injury has been reported following duloxetine use. This study further examines the hepatic safety of duloxetine in a large US health insurance database.

**Methods:**

In this propensity score-matched cohort analysis in a US commercially insured population (01 August 2004 to 31 December 2010), we compared individuals with depression and without liver disease who initiated duloxetine to comparators (venlafaxine or selective serotonin reuptake inhibitors [SSRIs], and individuals with pharmacologically untreated depression). We estimated incidence rates (IR) and 95 % confidence intervals (CI) for medical record-confirmed hepatic-related death, liver failure, and other clinically significant hepatic injury.

**Results:**

Among 30,844 duloxetine initiators, 21,000 were matched to venlafaxine initiators, 28,479 to SSRI initiators, and 22,714 to untreated patients. There were no cases of hepatic-related death or liver failure. IRs of other clinically significant hepatic injury without documented alternate etiologies were higher but not statistically significant among duloxetine initiators compared to initiators of venlafaxine (0.7/1000 person-years [PY] [95 % CI: 0.2 − 1.5] vs. 0.0/1000 PY [95 % CI: 0.0 − 0.3]) and SSRIs (0.4/1000 PY [95 % CI: 0.1 − 1.0] vs. 0.0/1000 PY [95 % CI: 0.0 − 0.3]). IRs were similar among duloxetine and untreated patients (0.5/1000 PY [95 % CI: 0.1 − 1.3] vs. 0.5/1000 PY [95 % CI: 0.1 − 1.5]). When hepatic outcomes were considered irrespective of alternate etiologies, similar results were observed.

**Conclusions:**

Our findings, while not statistically significant, may suggest a higher incidence of hepatic injury other than hepatic-related death or liver failure among duloxetine initiators compared to venlafaxine and possibly SSRIs, but not untreated patients. These differences remain consistent with chance, and an elevated risk cannot be ruled in or out.

## Background

Duloxetine (Cymbalta; Eli Lilly and Company, Indianapolis, IN, USA) is a serotonin-norepinephrine reuptake inhibitor (SNRI) approved in 2004, with indications for the treatment of major depressive disorder, diabetic peripheral neuropathic pain, fibromyalgia, generalized anxiety disorder, stress urinary incontinence (in European Union), and chronic pain states associated with chronic lower back pain and osteoarthritis (in some geographies).

Drug-induced liver injury (DILI) is the most frequent cause of acute liver failure in the United States (US) and a frequent reason for withdrawal of a drug from the market [1, 2]. Elevations of alanine aminotransferase to three times the upper limit of normal were observed in clinical trials in approximately 1 % of duloxetine-treated depressed recipients [3]. Hepatic injury with a hepatocellular, cholestatic, or mixed pattern has also been reported rarely in association with duloxetine during the post-marketing period [4, 5].

While DILI has been reported with several antidepressant agents [6, 7], information regarding the risk for liver injury in patients treated with duloxetine and comparisons with other antidepressants in routine clinical practice remains limited. A recent study by Xue et al. revealed that for hepatic injury of lesser severity such as elevated aminotransferases, duloxetine was associated with a higher incidence relative to the venlafaxine and non-depressed cohorts, but not relative to the selective serotonin reuptake inhibitor (SSRI) cohort [8]. However, for serious liver injury such as hepatic failure and hepatic-related death, the duloxetine and other antidepressant cohorts did not differ significantly and the overall hepatic safety profiles of duloxetine and SSRIs were similar, albeit with modest statistical power. The study by Xue et al., conducted within data from a commercial health plan, was restricted to the first 2 years following the launch of duloxetine in the US and included patients with and without baseline hepatic risks and active liver disease, complicating determinations of the association between duloxetine and liver injury. We performed a follow-up retrospective cohort study within the same database, with expanded accrual to include data from the first 6 years following the launch of duloxetine. To minimize the capture of non-drug-related liver injury and provide a specific focus on clinically significant potential drug-related liver injury, this study was restricted to subjects without pre-existing liver conditions. The objective of this study was to estimate the absolute and relative incidence of clinically significant hepatic events among patients with depression and without pre-existing liver disease who initiated duloxetine relative to each of the following propensity score-matched comparison cohorts: patients with depression who initiated venlafaxine, patients with depression who initiated SSRIs, and patients with a diagnosis of depression who did not receive treatment.

## Methods

### Data source and study population

Data included in this retrospective cohort study came from the Optum Research Database (ORD), an electronic health care database of a large US health plan including demographics, pharmacy use, and medical and facility claims, which provide dates on services, procedures, and their accompanying diagnoses. The population is geographically diverse and comprises approximately 3–4 % of the US population.

We included commercially insured health plan members 18 years of age or older, with complete medical coverage and pharmacy benefits, who:

- initiated a study antidepressant (duloxetine, venlafaxine, or an SSRI) between 01 August 2004 and 31 September 2010 (first dispensing following 12 months of continuous enrollment without similar dispensing) or who had an inpatient or outpatient physician diagnosis of depression (International Classification of Diseases, Ninth Revision [ICD-9], codes 311.x, 296.x, 309.x, or 300.x) without dispensings for an antidepressant medication
- had at least 12 months prior continuous enrollment criteria (baseline period);
- had a claim for diagnosis of depression during the 12-month baseline period.

We excluded members if they had a diagnosis for a hepatic condition during the baseline period (Table 1), or if they were located in the Louisiana and Mississippi regions with cohort entry date prior to 01 September 2006, as the availability of medical records during this period was likely to be affected by disruptions following Hurricane Katrina. Subjects who qualified as initiators of more than one study drug entered the cohort of the earliest initiated study drug. Cohort entry occurred on the date of dispensing of the cohort-defining antidepressant (treated cohorts) or an office visit associated with a claim with a depression diagnosis (pharmacologically untreated cohort).

**Table 1:** Hepatic conditions exclusion criteria, identified on the basis of health care claims

Condition	
Heart failure	
Thrombocytopenia	
Positive markers for hepatitis infections	
HIV infection	
Abnormal aminotransferases	
Abnormal total bilirubin	
Abnormal transferrin saturation	
Jaundice/icterus	
Acute or sub-acute necrosis of liver	
Veno-occlusive liver disease (not including Budd Chiari syndrome)	
Liver infarction	
Hepatic coma	
Hepatorenal syndrome	
Chronic liver disease, cirrhosis, and/or fibrosis	
Chronic hepatitis	
Chronic active hepatitis	
Acute hepatitis	
Alcoholic hepatitis	
Autoimmune hepatitis	
Viral hepatitis	
Unspecified or cryptogenic hepatitis	
Nonalcoholic hepatitis	
Biliary tract obstruction, stricture, stones	
Primary or metastatic neoplasia of the liver and hepatic ducts	
Primary or metastatic tumors elsewhere	
Hepatic encephalopathy	
Liver transplant	
Ascites	
Hepatectomy	
Other liver operations	
Hereditary hemochromatosis	
Disorders of copper metabolism (Wilson’s disease)	
Alpha-1 antitripsin deficiency	
Celiac disease	
Sclerosing cholangitis	
Primary biliary cirrhosis	
Liver helminth, fluke, parasite	
Budd-Chiari Syndrome	
Abdominal trauma	

Approval of the study protocol and waiver of patient authorization were obtained from the New England Institutional Review Board and affiliated Privacy Board.

### Exposure assessment

We defined exposure according to subjects’ initial cohort assignment (initiator of duloxetine, venlafaxine, or an SSRI, or patient with pharmacologically untreated depression). This “as-matched” exposure classification is analogous to “intent-to-treat” analyses used in randomized trials.

### Outcome assessment

The primary outcomes were cases of clinically significant hepatic injury, defined as 3 distinct categories informed by criteria in Chalasani et al. [9] and de Abajo [10] and 2 composite categories (Table 2):

**Table 2:** Hepatic outcome definitions

Hepatic outcome	Definition
Hepatic-related death	Fatality where the underlying or any of the contributory causes of death in the death certificate was a hepatic event based on linkage to the National Death Index (NDI), or in the absence of a death certificate, where there is evidence of an underlying hepatic event on the basis of health insurance claims in relation to the death
Liver failure (non-fatal), in the absence of explicit identification of an alternative etiology	Liver failure, not resulting in death, defined by the following conditions
	● Hepatic failure
	● Fulminant hepatitis (non-infectious)
	● Hepatic encephalopathy
	● Hepatic coma
	● Liver transplant or renal and liver transplant
	● When explicitly associated with acute liver disease:
	● Acquired coagulation factor V deficiency due to liver disease (factor V level <30 %)
	● Acquired hypoprothrombinemia
	● International normalized ratio (INR) >1.5, prothrombin time >16 s
Other clinically significant hepatic injury, in the absence of explicit identification of an alternative etiology	Hepatic injury, defined by any of the following:
	● ALT > 500 IU/ml or ≥10x ULN, with any of the following: incident toxic hepatitis; incident acute hepatitis; incident hepatic necrosis; incident toxic liver disease; or incident toxic hepatitis
	● ALT > 250 IU/mL or > 5x ULN with any of the following: nausea/vomiting; abdominal pain; weakness; or fatigue
	● ALT > 3x ULN with any of the following: jaundice/icterus when alkaline phosphatase <2x ULN; ascites; or a combination of total bilirubin >2x ULN (Hy’s rule) and alkaline phosphatase <2x ULN
Non-serious hepatic enzyme elevation, in the absence of explicit identification of an alternative etiology	Presence of the combination of all the following, provided the patient is asymptomatic:
	● 5x ULN < ALT <10x ULN
	● T bilirubin <2x ULN
	● Any ICD-9 hepatic code, validated by medical record review, when no symptoms associated with it and in absence of any codes for hepatic failure or serious hepatic injury (hepatic coma, jaundice, ascites, etc.)

- hepatic-related death
- liver failure
- other clinically significant hepatic injury
- hepatic-related death or liver failure combined
- all clinically significant hepatic outcomes combined.

The secondary outcome was non-serious asymptomatic hepatic enzyme elevations.

Potential cases were identified on the basis of ICD-9 diagnosis or procedure codes. An independent clinician blinded to drug exposure reviewed claims for each potential case and selected an appropriate provider to obtain medical records for those with claims consistent with the occurrence of a study outcome. Two independent academic hepatologists reviewed the medical records of each potential case and adjudicated case status attributing cases identified from any administrative code to the most severe category for which the event qualified. They identified the most severe hepatic event confirmed for the episode of care, determined whether an alternate etiology for a confirmed hepatic event was explicitly identified based on their expert clinical opinion, and identified the date of diagnosis based on the clinical data in the medical record. Discrepancies between the 2 reviewers were resolved by consensus. Hepatic deaths were confirmed through a combination of medical record review and linkage to the National Death Index (NDI) [11] through 31 December 2010. Deaths from hepatic conditions were identified by ICD-10 codes indicating hepatic disorders (B15-B19, K70-K77, B94.2, R17, R18) as a listed cause of death in the NDI.

### Covariate assessment and propensity score estimation

We ascertained a range of characteristics, including psychological comorbidities, hepatic comorbidities and risk factors, medications, and health care services, that were specified a priori and ascertained based on the presence of diagnoses, procedures, and pharmacy dispensings in the health care claims during the 12-month baseline. Additional baseline covariates were empirically identified by listing the 100 most frequent drug classes dispensed to the duloxetine initiator cohort during the baseline period, along with the most frequent diagnoses (at the 3-digit ICD-9 level) and procedures (based on Current Procedural Terminology (CPT) codes). These baseline characteristics were included in the propensity score analysis.

Duloxetine initiators were matched on a 1:1 ratio to each of the other 3 comparator cohorts (venlafaxine, SSRI, pharmacologically untreated) on the basis of estimated propensity scores, independently within calendar blocks of cohort accrual with the purpose of balancing the cohorts with respect to baseline characteristics [12–16]. Propensity scores were estimated using logistic regression models of exposure (duloxetine or comparator) on covariates, with a forced inclusion of variables identified *a priori*, and stepwise selection of remaining variables.

### Follow-up

We followed subjects from the day following cohort entry to the earliest of: discontinuation of study drug (or for the pharmacologically untreated cohort, initiation of a study drug); disenrollment from the health plan; occurrence of a study outcome event (followed separately for each outcome category); or 31 December 2010. Patients for whom the first claims-identified event was not confirmed via medical record review or for whom medical charts could not be obtained for review were censored on the date of the claims-identified event.

“Time to treatment discontinuation” was defined primarily as the time from cohort entry through 15 days following discontinuation of treatment based on dates of dispensing and days supply (primary definition). To assess the impact of varying the length of the observation window on estimated effects, two additional definitions were applied in secondary analyses: (1) time from cohort entry through 30 days following discontinuation of treatment; and (2) time restricted to the 1st 90 days following cohort entry. For patients in the untreated depression cohort, each patient was assigned a proxy “treatment discontinuation” date corresponding to the date of treatment discontinuation of their matched duloxetine comparator.

### Statistical analysis

#### Main analyses

Baseline characteristics were tabulated for the cohorts prior to matching and within the matched cohorts, using frequencies and proportions for categorical variables, and means and standard deviations for continuous variables. To assess whether potential imbalances remained after propensity score matching, absolute standardized differences were calculated for covariates included in the propensity score model [17, 18]; variables with an absolute standardized difference >0.1 after propensity score matching were identified as potential confounders for inclusion in multivariable outcome models.

Incidence rates of hepatic injury were calculated as the number of confirmed cases divided by the relevant person-time. We also estimated 95 % confidence intervals (CIs) of the IRs for the duloxetine initiator and comparator cohorts. Incidence rate ratios (IRR) and associated 95 % CIs comparing the rate of clinically significant hepatic injury among duloxetine initiators with each of the 3 comparator cohorts were estimated using exact methods.

We limited primary outcomes to cases of hepatic injury without explicit identification of an alternative etiology, and assessed the impact of excluding cases on the basis of alternate etiology through comparison to a secondary analysis in which all confirmed hepatic outcomes were included [19].

#### Sensitivity analyses

Insurance claims might incompletely capture covariates or study exclusions, especially those related to hepatic injury (e.g., abnormal liver function test results). We therefore assessed the completeness of covariate and exclusion capture through additional sampled medical record review. Characteristics relating to depression and hepatic risk factors, such as past hepatitis, alcohol use, and other data (e.g., liver function test results) were ascertained through review of medical records from around the time of cohort entry for 1260 randomly selected duloxetine initiators and propensity score-matched comparators (315 from each cohort).

To assess the potential for bias introduced by differential loss to follow-up, patients who were censored during follow-up were characterized according to their baseline characteristics, by matched cohort.

All analyses were conducted using SAS version 9.2 (SAS Institute, Inc., Cary, NC).

### Ethics approval

The New England Institutional Review Board approved this study, and the Privacy Board approved a waiver of authorization for access to medical records and for the NDI linkage.

## Results

### Study population

From 01 August 2004 to 30 September 2010, 30,844 initiators of duloxetine, 29,243 initiators of venlafaxine, 166,236 initiators of SSRI, and 311,338 subjects with pharmacologically untreated depression met eligibility criteria prior to propensity score matching (Table 3).

**Table 3:** Study eligibility and exclusions

	Duloxetine		Venlafaxine		SSRI		Untreated	
	N removed	N eligible remaining	N removed	N eligible remaining	N removed	N eligible remaining	N removed	N eligible remaining
Member of commercial health insurance plan, with medical coverage and pharmacy benefits, and presence of qualifying event^a^ during the accrual period		156,682 (100.0 %)		231,793 (100 %)		1,314,147 (100 %)		1,577,784 (100 %)
Baseline exclusions								
Less than 18 years at index date	1396	155,286 (99.1 %)	2482	229,311 (98.9 %)	33,476	1,280,671 (97.5 %)	60,552	1,517,232 (96.2 %)
Less than 12 months prior continuous enrollment	79,819	75,467 (48.2 %)	136,147	93,164 (40.2 %)	780,943	499,728 (38.0 %)	791,027	726,205 (46.0 %)
Prior dispensing of study drug or drug class in the baseline	0	75,467 (48.2 %)	23,753	69,411 (29.9 %)	134,294	365,434 (27.8 %)	334,496	391,709 (24.8 %)
Had no depression diagnosis in the baseline	27,596	47,871 (30.6 %)	24,844	44,567 (19.2 %)	153,173	212,261 (16.2 %)	0	391,709 (24.8 %)
Had hepatic injury/chronic hepatic condition in the baseline	10,592	37,279 (23.8 %)	9173	35,394 (15.3 %)	39,354	172,907 (13.2 %)	76,815	314,894 (20.0 %)
Region affected by Hurricane Katrina	489	36,790 (23.5 %)	509	34,885 (15.1 %)	2020	170,887 (13.0 %)	3556	311,338 (19.7 %)
Cohort assignment based on earliest qualifying index date (pre-propensity score matching)	30,844 (19.7 %)		29,243 (12.6 %)		166,236 (12.6 %)		311,338 (19.7 %)	

^a^Initiation of a study drug, or for untreated patients, a claim for depression associated with a physician visit

Of the duloxetine initiators, the following numbers were matched in a 1:1 ratio to each of the other groups: 21,000 (68.1 %) to venlafaxine initiators, 28,479 (92.3 %) to SSRI initiators, and 22,714 (73.6 %) to patients with a diagnosis of depression who did not receive an antidepressant. Conversely, 21,000 out of 29,243 (71.8 %) venlafaxine initiators, 28,479 out of 166,236 (17.1 %) SSRI initiators, and 22,714 out of 311,338 (7.3 %) patients in the untreated cohort were matched to duloxetine initiators.

### Descriptive baseline characteristics

Prior to propensity score matching, duloxetine initiators were more likely to be older compared to the other 3 comparator cohorts, and were more likely to be female compared to the SSRI and pharmacologically untreated depression cohorts. Duloxetine initiators also had a higher prevalence a wide range of a priori-defined characteristics representing baseline neuropsychological comorbidities, hepatic risk factors, medication use, and health services utilization.

Matching on propensity score resulted in matched duloxetine initiator and comparator cohorts that were well-balanced for most measured baseline characteristics retained in the final propensity score models (Table 4). Standardized difference scores did not exceed the threshold of >0.1 for any of the variables included in the propensity scores.

**Table 4:** Baseline characteristics among duloxetine and comparator propensity score-matched cohorts, ORD, 01 August 2004–30 September 2010

Baseline Characteristic	Duloxetine-Venlafaxine				Duloxetine-SSRI				Duloxetine-Untreated			
	Duloxetine		Venlafaxine		Duloxetine		SSRIs		Duloxetine		Untreated	
	(*N* = 21,000)		(*N* = 21,000)		(*N* = 28,479)		(*N* = 28,479)		(*N* = 22,714)		(*N* = 22,714)	
	N	%	N	%	N	%	N	%	N	%	N	%
Age group (years)												
18–25	1855	8.8	1807	8.6	2154	7.6	2057	7.2	1829	8.1	1812	8.0
26–30	1748	8.3	1758	8.4	2070	7.3	1992	7.0	1746	7.7	1764	7.8
31–35	2473	11.8	2487	11.8	3101	10.9	3128	11.0	2493	11.0	2501	11.0
36–40	3067	14.6	2979	14.2	4034	14.2	4008	14.1	3160	13.9	3108	13.7
41–50	6152	29.3	6218	29.6	8667	30.4	8733	30.7	6725	29.6	6703	29.5
51–60	4371	20.8	4419	21.0	6390	22.4	6436	22.6	5041	22.2	5030	22.1
61–70	1176	5.6	1175	5.6	1817	6.4	1871	6.6	1506	6.6	1582	7.0
≥ 71	158	0.8	157	0.7	246	0.9	254	0.9	214	0.9	214	0.9
Female	15,074	71.8	15,092	71.9	20,570	72.2	20,405	71.6	15,974	70.3	16,020	70.5
Number of unique ICD-9 codes												
0–4	1924	9.2	1948	9.3	2291	8.0	2169	7.6	2019	8.9	1894	8.3
5–8	4228	20.1	4208	20.0	5002	17.6	5006	17.6	4270	18.8	4145	18.2
9–12	4603	21.9	4657	22.2	5893	20.7	6039	21.2	4874	21.5	4866	21.4
13–16	3706	17.6	3704	17.6	5059	17.8	5086	17.9	4071	17.9	4092	18.0
17+	6539	31.1	6483	30.9	10,234	35.9	10,179	35.7	7480	32.9	7717	34.0
Number of different drugs dispensed												
0–3	2525	12.0	2410	11.5	2656	9.3	2253	7.9	2640	11.6	2482	10.9
4–7	6365	30.3	6427	30.6	7553	26.5	7654	26.9	6890	30.3	6873	30.3
8+	12,110	57.7	12,163	57.9	18,270	64.2	18,572	65.2	13,184	58.0	13,359	58.8
Total costs (mean std)	7944.9	14,910.5	7685.7	14,071.0	9354.0	16,773.2	9113.6	18,729.4	8295.0	15,748.1	8201.4	15,713.5
ᅟ	ᅟ	ᅟ	ᅟ	ᅟ	ᅟ	ᅟ	ᅟ	ᅟ	ᅟ	ᅟ	ᅟ	ᅟ
Psychotic disorders	7746	36.9	7685	36.6	11,187	39.3	11,029	38.7	7345	32.3	7349	32.4
Fibromyalgia	1936	9.2	1858	8.8	3760	13.2	3625	12.7	2610	11.5	2620	11.5
Back pain	2974	14.2	2863	13.6	4916	17.3	4921	17.3	3658	16.1	3764	16.6
Bilirubin test	971	4.6	972	4.6	1364	4.8	1350	4.7	1042	4.6	1031	4.5
ALT/AST/ALP test	788	3.8	790	3.8	1205	4.2	1208	4.2	924	4.1	952	4.2
NSAID use (excluding diclofenac)	5643	26.9	5730	27.3	8573	30.1	8591	30.2	6534	28.8	6776	29.8
Use of other medications												
Diclofenac	736	3.5	710	3.4	1206	4.2	1203	4.2	900	4.0	920	4.1
Valproic acid	408	1.9	439	2.1	653	2.3	673	2.4	451	2.0	471	2.1
Nitrofurantoin	796	3.8	773	3.7	1146	4.0	1121	3.9	875	3.9	962	4.2
Fluconazole	1684	8.0	1701	8.1	2428	8.5	2417	8.5	1872	8.2	1974	8.7
Statins	3008	14.3	3002	14.3	4518	15.9	4666	16.4	3584	15.8	3609	15.9
Anticonvulsants	5403	25.7	5406	25.7	8817	31.0	8745	30.7	5799	25.5	5774	25.4
Anxiolytics or sedative hypnotics	10,192	48.5	10,072	48.0	14,698	51.6	14,701	51.6	10,673	47.0	10,724	47.2
Antihistamines	4364	20.8	4315	20.5	6230	21.9	6183	21.7	4699	20.7	4920	21.7
Antipsychotics	1689	8.0	1710	8.1	2549	9.0	2510	8.8	1455	6.4	1773	7.8
Narcotic analgesics	9533	45.4	9452	45.0	14,366	50.4	14,339	50.3	10,923	48.1	11,090	48.8

*ORD* Optum Research Database, *SSRI* selective serotonin reuptake inhibitor, *ALT* alanine aminotransferase, *AST* aspartate aminotransferase, *ALP* alkaline phosphatase, *NSAID* nonsteroidal anti-inflammatory drug, *ICD-9* International Classification of Diseases, 9th Edition, *std* standard deviation

### Identification and confirmation of hepatic events

There were 969 potential cases of clinically significant hepatic injury for 962 individuals among the propensity score-matched cohorts (nine hepatic-related deaths, 32 liver failure events, and 928 other clinically significant hepatic injury events). Of these, 74 % (716) of charts (six hepatic-related deaths, 25 liver failure events, and 685 other clinically significant hepatic injury events) representing 712 individuals were available for abstraction and review by the clinical consultants.

Fifty-four of the 716 abstracted potential cases (representing 53 unique events) were confirmed as a clinically significant hepatic injury or a non-serious asymptomatic hepatic enzyme elevation. An alternate etiology was identified for 35 of the 54 hepatic events; these included cases adjudicated as having documentation of acetominophen toxicity, cholelithiasis with or without pancreatitis, liver injury following motor vehicle accident, sepsis, hepatitis C virus, Epstein Barr virus, fatty liver disease, alcohol-related injury, hypotension, other medications, pancreatic cancer, and lymphoma. The remaining 18 events (zero hepatic-related death, zero liver failure, 12 other clinically significant hepatic injury, six non-serious hepatic enzyme elevation) were adjudicated as possible or definite drug-associated liver injury.

When observation was restricted to the primary follow-up window (time to treatment discontinuation plus 15 days), 41 unique confirmed events remained, of which 11 were adjudicated as possible or definite drug-associated liver injury (zero hepatic-related death, zero liver failure, eight other clinically significant hepatic injury, three non-serious enzyme elevation).

One case in the duloxetine cohort was initially confirmed by independent adjudicators as a hepatic injury event without alternate etiology, but after completion of the analyses, a further review of the health care claims occurring within 45 days prior to adjudicated event date for this case indicated the presence of diagnosis codes in the claims data consistent with alternate etiologies of acute viral infection and chronic liver disease, cirrhosis, or fibrosis. We conducted an additional clinical review of the medical record for this case. In the additional review, the reviewers re-confirmed the case was a hepatic injury event, but contrary to their initial assessment, adjudicated the case as a confirmed hepatic injury event *with alternate etiology*. As this posthoc review of the single case occurred several months following the initial main review and was undertaken outside of the protocol, all analyses presented are based on data from the protocol-specified clinical review only.

### Incidence of liver injury

Considering cases of hepatic injury without an alternate etiology, zero cases of hepatic-related death and zero cases of liver failure were confirmed (Table 5). For the outcome, other clinically significant hepatic injury, the IR among duloxetine initiators was 0.7 per 1000 person-years (95 % CI: 0.2, 1.5) among those matched to venlafaxine compared to 0.0 per 1000 person-years (95 % CI: 0.0, 0.3) among venlafaxine initiators, and was 0.4 per 1000 person-years (95 % CI: 0.1, 1.0) among those matched to SSRI compared to 0.0 per 1000 person-years (95 % CI: 0.0, 0.3) among SSRI initiators. IRs were similar among duloxetine initiators and the matched untreated cohort (0.5 per 1000 person-years [95 % CI: 0.1, 1.3] among duloxetine initiators vs. 0.5 per 1000 person-years [95 % CI: 0.1, 1.5] among untreated comparators). For the outcome, non-serious hepatic enzyme elevations, a total of 3 events were confirmed among the study cohorts (1 duloxetine, 2 SSRI), corresponding to an IR of 0.1 per 1000 person-years (95 % CI: 0.0, 0.5) among duloxetine initiators and an IR of 0.2 per 1000 person-years (95 % CI: 0.0, 0.7) among SSRI initiators. Varying the length of the exposure (through restriction to the 1st 90 days following cohort entry, or expansion to include time on therapy through 30 days following treatment discontinuation) produced substantively similar results as the primary analysis (data not shown).

**Table 5:** IRs, IRRs, and 95 % confidence intervals of hepatic events without identified alternate etiology, follow-up through 15 days following treatment discontinuation, matched duloxetine and comparator cohorts, ORD, 01 August 2004 to 31 December 2010

Outcome	Cohort	Person-Years	No. Cases	IR	95 CI %	IRR	95 % CI
Hepatic-related death	Duloxetine	7633.5	0	0.0	(0.0, 0.4)	NA	NA
	Venlafaxine	8838.7	0	0.0	(0.0, 0.3)		
	Duloxetine	10,411.9	0	0.0	(0.0, 0.3)	NA	NA
	SSRI	9835.6	0	0.0	(0.0, 0.3)		
	Duloxetine	8116.7	0	0.0	(0.0, 0.4)	NA	NA
	Untreated	5966.1	0	0.0	(0.0, 0.5)		
Hepatic failure	Duloxetine	7631.7	0	0.0	(0.0, 0.4)	NA	NA
	Venlafaxine	8836.6	0	0.0	(0.0, 0.3)		
	Duloxetine	10,410.6	0	0.0	(0.0, 0.3)	NA	NA
	SSRI	9833.9	0	0.0	(0.0, 0.3)		
	Duloxetine	8116.2	0	0.0	(0.0, 0.4)	NA	NA
	Untreated	5965.8	0	0.0	(0.0, 0.5)		
Other clinically significant hepatic injury^a^	Duloxetine	7548.5	5	0.7	(0.2, 1.5)	undef.	(1.1, inf.)
	Venlafaxine	8745.2	0	0.0	(0.0, 0.3)		
	Duloxetine	10,300.9	4	0.4	(0.1, 1.0)	undef.	(0.6, inf.)
	SSRI	9753.2	0	0.0	(0.0, 0.3)		
	Duloxetine	8035.8	4	0.5	(0.1, 1.3)	1.0	(0.2, 6.7)
	Untreated	5931.9	3	0.5	(0.1, 1.5)		
Hepatic-related death and liver failure combined	Duloxetine	7631.7	0	0.0	(0.0, 0.4)	NA	NA
	Venlafaxine	8836.6	0	0.0	(0.0, 0.3)		
	Duloxetine	10,410.6	0	0.0	(0.0, 0.3)	NA	NA
	SSRI	9833.8	0	0.0	(0.0, 0.3)		
	Duloxetine	8116.2	0	0.0	(0.0,0.4)	NA	NA
	Untreated	5965.6	0	0.0	(0.0, 0.5)		
All clinically significant hepatic categories combined	Duloxetine	7548.5	5	0.7	(0.2, 1.5)	undef.	(1.1, inf.)
	Venlafaxine	8745.2	0	0.0	(0.0, 0.3)		
	Duloxetine	10,300.9	4	0.4	(0.1, 1.0)	undef.	(0.6, inf.)
	SSRI	9753.2	0	0.0	(0.0, 0.3)		
	Duloxetine	8035.8	4	0.5	(0.1, 1.3)	1.0	(0.2, 6.7)
	Untreated	5931.9	3	0.5	(0.1, 1.5)		
Non-serious hepatic enzyme elevation	Duloxetine	7548.5	1	0.1	(0.0, 0.7)	undef.	(0.0, inf.)
	Venlafaxine	8745.2	0	0.0	(0.0,0.3)		
	Duloxetine	10,300.9	1	0.1	(0.0, 0.5)	0.5	(0.0, 9.1)
	SSRI	9753.0	2	0.2	(0.0, 0.7)		
	Duloxetine	8035.8	1	0.1	(0.0, 0.7)	undef.	(0.0, inf.)
	Untreated	5932.0	0	0.0	(0.0, 0.5)		

*ORD* Optum Research Database, *IR* incidence rate, representing number of events per 1000 person-years, *CI* confidence interval, *IRR* incidence rate ratio, *NA* not available, *undef*.undefined, *inf.* infinity

^a^One case of other clinically significant hepatic injury in the duloxetine cohort was initially confirmed by independent adjudicators as a confirmed case without alternate etiology. However, following a *post hoc* review by the adjudicators, this case was classified as a hepatic injury *with* alternate etiologies. Because we conducted this *post hoc* review outside of the protocol, the case remains in these data as a confirmed case without alternate etiology

When all confirmed hepatic outcomes were considered irrespective of alternate etiologies, similar results were observed (Table 6). Duloxetine initiators had an IR suggestive of elevated risk for all clinically significant hepatic injuries combined when compared with initiators of venlafaxine (IRR: 3.2; exact 95 % CI: 0.9, 13.7). Smaller elevations were observed comparing duloxetine initiators to initiators of SSRIs (IRR: 1.3; exact 95 % CI: 0.5, 3.3), while IRs observed among duloxetine initiators and the untreated cohort were similar (IRR: 1.0; exact 95 % CI: 0.4, 2.9).

**Table 6:** IRs, IRRs, and 95 % confidence intervals of hepatic events irrespective of determination of alternate etiology, follow-up through 15 days following treatment discontinuation, matched duloxetine and comparator cohorts, ORD, 01 August 2004 to 31 December 2010

Outcome	Cohort	Person-Years	No. Cases	IR	95 % CI	IRR	95 % CI
Hepatic-related death	Duloxetine	7633.5	0	0.0	(0.0, 0.4)	NA	NA
	Venlafaxine	8838.7	0	0.0	(0.0, 0.3)		
	Duloxetine	10,411.9	0	0.0	(0.0, 0.3)	NA	NA
	SSRI	9835.6	0	0.0	(0.0, 0.3)		
	Duloxetine	8116.7	0	0.0	(0.0, 0.4)	NA	NA
	Untreated	5966.1	0	0.0	(0.0, 0.5)		
Hepatic failure	Duloxetine	7631.7	0	0.0	(0.0, 0.4)	NA	NA
	Venlafaxine	8836.6	0	0.0	(0.0, 0.3)		
	Duloxetine	10,410.6	0	0.0	(0.0, 0.3)	NA	NA
	SSRI	9833.9	0	0.0	(0.0, 0.3)		
	Duloxetine	8116.2	0	0.0	(0.0, 0.4)	NA	NA
	Untreated	5965.8	0	0.0	(0.0, 0.5)		
Other clinically significant hepatic injury	Duloxetine	7548.5	11	1.5	(0.7, 2.6)	3.2	(0.9, 13.7)
	Venlafaxine	8745.2	4	0.5	(0.1, 1.2)		
	Duloxetine	10,300.9	14	1.4	(0.7, 2.3)	1.3	(0.5, 3.3)
	SSRI	9753.2	10	1.0	(0.5,1.9)		
	Duloxetine	8035.8	11	1.4	(0.7, 2.4)	1.0	(0.4, 2.9)
	Untreated	5931.9	8	1.3	(0.6, 2.7)		
Hepatic-related death and liver failure combined	Duloxetine	7631.7	0	0.0	(0.0,0.4)	NA	NA
	Venlafaxine	8836.6	0	0.0	(0.0, 0.3)		
	Duloxetine	10,410.6	0	0.0	(0.0, 0.3)	NA	NA
	SSRI	9833.8	0	0.0	(0.0, 0.3)		
	Duloxetine	8116.2	0	0.0	(0.0, 0.4)NA		NA
	Untreated	5965.6	0	0.0	(0.0, 0.5)		
All clinically significant hepatic categories combined	Duloxetine	7548.5	11	1.5	(0.7, 2.6)	3.2	(0.9, 13.7)
	Venlafaxine	8745.2	4	0.5	(0.1, 1.2)		
	Duloxetine	10,300.9	14	1.4	(0.7, 2.3)	1.3	(0.5, 3.3)
	SSRI	9753.2	10	1.0	(0.5, 1.9)		
	Duloxetine	8035.8	11	1.4	(0.7, 2.4)	1.0	(0.4, 2.9)
	Untreated	5931.9	8	1.3	(0.6, 2.7)		
Non-serious hepatic enzyme elevation	Duloxetine	7548.5	1	0.1	(0.0, 0.7)	undef.	(0.0,inf.)
	Venlafaxine	8745.2	0	0.0	(0.0, 0.3)		
	Duloxetine	10,300.9	1	0.1	(0.0, 0.5)	0.5	(0.0,9.1)
	SSRI	9753.0	2	0.2	(0.0, 0.7)		
	Duloxetine	8035.8	1	0.1	(0.0, 0.7)	0.7	(0.0,57.9)
	Untreated	5932.0	1	0.2	(0.0, 0.9)		

ORD Optum Research Database, *IR* incidence rate, representing number of events per 1000 person-years, *CI* confidence interval, IRR: rate ratio, *NA* not available, *undef.* undefined, *inf.* infinity

### Sensitivity analyses

#### Medical record validation of baseline comparability

From the 315 individuals randomly selected from each of the duloxetine and comparator cohorts, a total of 213 charts (68 %), 212 charts (67 %), 223 charts (71 %), and 212 charts (67 %) were abstracted for the duloxetine, venlafaxine, SSRI, and untreated cohorts, respectively.

Compared to venlafaxine initiators, alcohol use/abuse was less frequently documented among the matched duloxetine initiators (5.1 % vs. 8.5 %), while current smoking was more frequently documented among duloxetine initiators (21.5 % vs. 16.5 %). Approximately 3.8 % of duloxetine initiators and 4.7 % of venlafaxine initiators had a record of abnormal liver enzymes, while 1.3 % of duloxetine initiators and 0.5 % of venlafaxine initiators had chronic liver disease documented in the medical records.

Compared to SSRI initiators with abstracted medical records, matched duloxetine initiators had a higher baseline prevalence of current smoking (20.7 % vs. 11.2 %), obesity (21.2 % vs. 18.8 %), and alcohol use/abuse (6.4 % vs. 3.1 %). SSRI and duloxetine initiators had a similar prevalence of documented abnormal liver enzymes (3.5 % vs. 3.6 %) and chronic liver disease (1.5 % vs. 0.9 %).

Compared to untreated individuals with abstracted medical records, duloxetine initiators were slightly more likely to have documented alcohol use/abuse (6.7 % vs. 3.8 %) and a record of abnormal liver enzymes (4.7 % vs. 2.8 %).

Similar patterns of attrition were observed across the study cohorts, with censoring of between 54-59 % of subjects in the duloxetine, venlafaxine, and SSRI cohorts within the first 8 weeks following initiation of the study drugs and 47 % of subjects in the untreated cohort within the same time frame. Comparison of these patients to the overall duloxetine and matched comparator cohorts suggests that patients who dropped out at any time during study follow-up had similar baseline characteristics as the overall matched cohorts (unpublished).

## Discussion

In our study, no confirmed cases of hepatic-related death or hepatic failure were identified. We found that duloxetine initiators had a statistically non-significant higher incidence rate of other clinically significant hepatic injuries compared to initiators of venlafaxine, and a more modest elevation compared to initiators of SSRIs. These differences are consistent with a hypothesis of similar outcome among initiators of duloxetine and the treated comparator study drugs, and an elevated risk cannot be ruled in or out. No differences in the incidence of clinically significant hepatic outcomes were observed in the duloxetine and untreated comparator cohorts.

Potential hepatic injuries, such as elevated hepatic enzyme levels have been reported for duloxetine in pre-marketing clinical trials and in the post-marketing period [6], but these reports have, in some cases, been associated with excessive alcohol use or pre-existing liver disease. The IRs of clinically significant hepatic events observed in our study are comparable or slightly lower than those observed in the earlier safety study of duloxetine conducted in the Optum Research Database [8]. The earlier assessment using the Optum Research Database found higher incidence rates of hepatic injury of lesser severity among duloxetine users relative to propensity score-matched venlafaxine users, but not relative to SSRIs. Our study – which expanded accrual to include data from the first 6 years following the launch of duloxetine and was restricted to patients without baseline hepatic disorders as identified using the health care claims – provides additional information suggestive of an increased incidence of hepatic events of lesser severity among duloxetine initiators relative to clinically relevant comparators. The IRs observed in our study are lower (albeit within the confidence limits) compared with estimated IRs reported in a study by Shin et al., [20] which identified and evaluated the incidence and outcomes of drug-associated liver injury using electronic medical record data and that similarly restricted the study population to patients without evidence of pre-existing liver disease or other comorbidity to minimize capture of non-drug-related liver injury.

This study has several limitations and considerations. The statistical power of this study to evaluate relative risks was limited due to the rarity of these outcomes. We observed no cases of hepatic-related death or liver failure in the duloxetine or the three comparator cohorts, and this study does not demonstrate an association with duloxetine use. Previously published estimates suggest liver injury potentially associated with duloxetine is infrequent [4, 20]; our study (comprising 7500 to 10,000 person-years of duloxetine exposure) had limited power to identify clinically significant injury between study groups. While assessments of whether risk for liver injury is modified by the presence of underlying liver disease is an important clinical question, the primary interest of this study was to evaluate potential for increased risk in a more homogeneous population of patients in terms of baseline risk, as follow on to that reported by Xue et al.[8]. To minimize the capture of non-drug-related liver injury and provide a specific focus on clinically significant potential drug-related liver injury, this study was restricted to subjects without pre-existing liver conditions. The objective of this study was to estimate the absolute and relative incidence of clinically significant hepatic events among patients with depression and without pre-existing liver disease who initiated duloxetine relative to each of the following propensity score-matched comparison cohorts: patients with depression who initiated venlafaxine, patients with depression who initiated SSRIs, and patients with a diagnosis of depression who did not receive treatment. In addition, we followed patients for the occurrence of liver injury during the time patients were actively on treatment (plus 15 days after treatment cessation), further limiting statistical power. An assessment of potential chronic liver injury could also improve power, but falls outside the scope of our study. As an observational study, patients who received duloxetine may differ from those who received a comparator drug or those who did not receive any pharmacological treatment for depression. If these differences are associated with hepatic injury, then the comparison of the duloxetine initiators to comparators may be biased. Duloxetine initiators and their comparators were not matched directly on specific baseline characteristics. Instead, this study used propensity score techniques to match a comparator group to the exposed cohort and address potential confounding by numerous covariates that are integrated into this single variable; comparison of the duloxetine initiators to the comparator cohorts suggested the cohorts were well-balanced for the claims-based covariates included in the propensity score model. The challenge of identifying potential cases of drug-induced liver injury on the basis of ICD-9 codes in terms of sensitivity and specificity has been noted previously [21, 22]. In our study, we sought to procure and review medical records for all potential cases to verify the occurrence of a drug-induced liver injury event, and the low yield of confirmed cases from our chart validation is consistent with expectation. Because we were not able to obtain all of the medical records sought (74 % obtained), some study events could not be confirmed. This led to reduced statistical power and reduced estimates of the absolute incidence rates. However, the proportion of medical records obtained was similar across the study cohorts, with the expectation that the comparisons were not differentially affected and therefore should be unbiased.

While balanced on claims-based covariates, information obtained from the medical records in the baseline validation sample suggests that duloxetine initiators may have differed from the comparator cohorts on certain characteristics that may not be adequately captured using the health care claims (e.g., smoking, pain-related syndromes, alcohol use/abuse). Medical record documentation of important potential confounders may itself be incomplete or differentially captured (e.g., alcohol use or abuse). Residual confounding by covariates not available from medical records is also possible. However, the balancing of measured characteristics achieved by propensity score limits the extent of the confounding that is possible. The US product label of duloxetine includes hepatotoxicity in the “Warnings and Precautions” section and warns against prescribing this drug to patients with substantial alcohol use or evidence of chronic liver disease [23]. If heightened awareness of the risk for hepatotoxicity led clinicians to conduct more comprehensive assessments when deciding to prescribe duloxetine or as a part of monitoring therapy, the resulting surveillance bias may have led to differential rates of diagnosis of hepatic injury. This study was restricted to a commercially insured population of patients without pre-existing hepatic conditions, and results may not be generalizable to the general patient populations who ultimately may use the drug.

This study made use of propensity score methods to account for potential confounding and further mitigated potential confounding from the presence of pre-existing hepatic disease through restriction of the study population to patients without baseline hepatic disorders (on the basis of the health care claims). In addition, this study focused on clinically significant potentially drug-associated liver injury events, validated by medical record review, occurring while exposed to a drug of interest or up to 15 days after last drug availability to evaluate the risk of idiosyncratic drug-induced liver injury at the typical time of its occurrence. By supplementing covariate ascertainment through medical record review, this study was able to assess the potential role of unmeasured confounding as a plausible explanation for the observed associations. Study outcomes were adjudicated through medical record review to ensure that the observed cases of hepatic injury actually occurred and were not simply rule-out diagnoses in an administrative database. The broad initial screen for potential cases coupled with clinical adjudication addresses both the sensitivity and the specificity of case identification.

Even with this rigorous assessment of study outcomes, one case of other clinically significant hepatic injury in the duloxetine cohort was initially confirmed by the independent adjudicators as a confirmed case *without* alternate etiology. However, following a *post hoc* review by the adjudicators, this case was classified as a hepatic injury *with* alternate etiologies. This subsequent finding suggests that there is some variability in determination of alternate etiology by the same reviewer over time, at least for this case. The additional medical record review was prompted by the observation that this case had claims consistent with alternate etiologies. The re-review was not applied systematically to all potential cases and, therefore, not all cases had the same opportunity to receive an alternate designation.

We chose not to conduct a re-review of all cases, and the analyses presented were based on the results of the initial case adjudication. There could be no guarantee that a second review would be more accurate than the initial review. Indeed, a small risk of misclassification is inherent when humans are involved in the adjudication process. This misclassification is, in expectation, non-differential with respect to exposure because we blinded the reviewed medical records with respect to exposure status. Given the limited statistical power of the study, despite any modifications to case classification or analyses, the study data would remain imprecise.

## Conclusions

This study observed no cases of hepatic-related death or liver failure in initiators of duloxetine or the 3 comparator cohorts, and this study does not demonstrate an association with duloxetine use. Applying a rigorous methodology that addresses confounding and outcome certainty, our findings while statistically not significant, may suggest an elevated IR of other clinically significant hepatic injury among duloxetine initiators compared to initiators of venlafaxine and possibly initiators of SSRIs, but not relative to pharmacologically untreated patients.

## Abbreviations

CI: Confidence interval
CPT: Current Procedural Terminology
DILI: Drug-induced liver injury
ICD-9: International Classification of Diseases, ninth revision
NDI: National Death Index
IR: Incidence rate
IRR: Incidence rate ratios
ORD: Optum Research Database
PY: Person-years
SNRI: serotonin-norepinephrine reuptake inhibitor
SSRI: selective serotonin reuptake inhibitor
US: United States
